# Tackling Skeletal Muscle Cells Epigenome in the Next-Generation Sequencing Era

**DOI:** 10.1155/2012/979168

**Published:** 2012-06-03

**Authors:** Raffaella Fittipaldi, Giuseppina Caretti

**Affiliations:** Department of Biomolecular Sciences and Biotechnology, University of Milano, Via Celoria 26, 20133 Milan, Italy

## Abstract

Recent advances in high-throughput technologies have transformed methodologies employed to study cell-specific epigenomes and the approaches to investigate complex cellular phenotypes. Application of next-generation sequencing technology in the skeletal muscle differentiation field is rapidly extending our knowledge on how chromatin modifications, transcription factors and chromatin regulators orchestrate gene expression pathways guiding myogenesis. Here, we review recent biological insights gained by the application of next-generation sequencing techniques to decode the epigenetic profile and gene regulatory networks underlying skeletal muscle differentiation.

## 1. Introduction

 During developmental processes, in response to external cues, changes in chromatin involving activation and repression of transcription factors and chromatin regulators (e.g., chromatin-modifying enzymes) underlie commitment of specific cells to defined lineages. Importantly, transcription factors and chromatin modifiers are also able to alter the cellular expression program to maintain cell identity even upon removal of the initiating differentiation stimuli [1]. During development and in adulthood, cellular identity is established and maintained by finely tuned mechanisms of gene repression and expression, preserved through rounds of cell division. Differentiation processes are achieved by sculpting cell-specific epigenomes, which establish and maintain cellular diversity [2]. Epigenetic information relies on several elements, such as histone modifications, histone variants, nucleosome accessibility, DNA methylation and hydroxymethylation, and nuclear organization. These chromatin states influence access of transcription factors and enzymes to the underlying DNA [1].

## 2. Epigenetic Regulators of Myogenesis

 Myogenic lineage commitment and execution of the terminal differentiation program relies on the activity of the paired-box transcription factors Pax3 and Pax7 and of the muscle regulatory factors (MRFs) MyoD, Myf5, MRF4, and myogenin [3]. Myogenesis is a multistep process, which restricts cell fate and commits cells to become skeletal muscle. During embryonic development, myogenic precursor cells derived from the somites express Pax3 and Pax3/Pax7 and are capable of proliferation and self-renewal [4, 5].

 In response to extrinsic signals, Pax3+ cells are committed to myoblasts and undergo terminal myogenic differentiation through the transcriptional network activated by MRFs. During postnatal life, muscle growth relies on satellite cells, which are a subpopulation of somite-derived cells that reside between myofibers and the basal lamina [5]. Satellite cells adopt a quiescent state, and upon environmental cues, such as mechanical stress, injury or in pathological environment of degenerative muscle diseases, they are activated to proliferate and terminally differentiate to regenerate muscle [3]. Satellite cells encompass a population of cells that maintains the uncommitted state and another group of cells that are committed to the myogenic lineage and will undergo myogenic differentiation. Because of these two distinct features, satellite cells are defined as bona fide adult stem cells.

 MRFs achieve the task of transcriptional activation through the participation of nonmuscle restricted transcriptional activators, such as the Mef2, Six, and Runx family members [6]. The progressive elucidation of transcription factors involved in myogenesis has revealed the strong contribution of several epigenetic regulators of skeletal muscle genes transcriptional activation: chromatin modifiers such as histone acetyltransferases and a subset of histone demethylases modify histones, promoting transcription activation. Additionally, chromatin remodeling complexes facilitate nucleosome mobility to favor the access of transcription factors to chromatin regulatory regions. At the onset of differentiation, histone acetyltransferases such as p300 and PCAF are recruited to muscle specific genes by myogenic bHLH, Mef2, and SRF proteins and exert their enzymatic activity on regulatory chromatin regions and on transcription factors such as MyoD [7, 8]. Moreover, the WDR5/Ash2L/MLL2 complex is engaged to the chromatin of Myf5 and myogenin genes by Pax7 and Mef2D, respectively, to methylate histone H3 at lysine 4 and to stimulate transcription activation [9, 10].

 Moreover, other sets of epigenetic regulators exert their function by repressing gene transcription: HDACs (e.g., SIRT1 and HDAC1/3/4/5) [8] and histone methylases such as Suv39h1 and the Polycomb repressive complex 2 (PRC2) complex are recruited at inactive muscle specific genes [3, 8]. Although HDACs role in regulating transcription has been traditionally viewed as mutually exclusive to HATs function, a novel model is emerging in which HDACs and HATs cooccupy active genes [11]. HDACs task on transcribed regions is to remove acetyl groups previously added by HATs and to reset the chromatin modification state in preparation of the next round of transcription. According to this model, HATs and HDACs activities are interconnected by a dynamic interplay in regulating gene transcription [11]. Although further investigation at the genomewide level is warranted to define HATs/HDACs interplay throughout myogenesis, the knowledge that MyoD, PCAF, and the histone deacetylase Sir2 are able to form a complex and associate with chromatin [12] hints for a dynamic role of HATs and HDACs in skeletal muscle specification and maintenance, as well as in their nonhistone targets modulation. Polycomb group (PcG) proteins are a set of proteins that play a prominent role in the maintenance of cellular identity and in the regulation of developmental genes. These specialized proteins assemble in different Polycomb Repressive Complexes (PRCs) that exert their repressive function by establishing a nonpermissive chromatin structure [13]. Ezh2, Suz12, and EED are the core components of PRC2, which is responsible for the di- and tri-trimethylation of histone H3 at lysine 27. This repressive mark subsequently confers to chromatin the ability to recruit the PRC1 complex that in turn catalyzes the ubiquitination of histone H2A and further compacts chromatin [14, 15]. PcGs contribution to the preservation of cell identity is achieved through the regulation of developmental genes from different lineages, both in embryonic and in lineage-committed stem cells [16]. In embryonic stem cells, PRC2 localizes to chromatin regulatory loci of developmental regulators often coexisting with H3K4 trimethylation, a histone mark associated with a transcriptionally permissive chromatin state [17–19].

 Several lines of evidence showed that enhancer of Zeste homolog 2 (Ezh2), the catalytic subunit of the PRC2 complex, is recruited to a subset of muscle-specific promoters in myoblasts [15, 20, 21] to prevent premature transcription, and it is displaced from these loci throughout the differentiation process. Furthermore, Juan et al. dissected the role of PRC2 in skeletal muscle stem cells, by exploiting satellite cells derived from mice with a Pax7-dependent Ezh2 deletion. The authors conclude that Ezh2 is essential for satellite cells self-renewal, proliferation, and cell identity. Deletion of Ezh2 leads to increased levels of the cell cycle inhibitor p16 (Cdkn2a), which affects satellite cells proliferation rate. Moreover, Ezh2-null satellite cells misexpress nonmyogenic lineage genes, normally repressed by PRC2 in skeletal satellite cells [22].

In summary, PcG role in satellite cells is to prevent ectopic differentiation, thus maintaining quiescence and proliferation states and safeguarding the mis-expression of non-myogenic genes in skeletal muscle cells. Another layer of control in myogenesis and muscle homeostasis is exerted by microRNAs (miRNAs) and long noncoding RNAs (lncRNAs) [23, 24]. miRNAs attenuate transcripts level through translation repression or mRNA degradation. Several miRNAs that modulate myogenesis have been shown to impact numerous processes in various lineages, such as miR-214 and miR-26a that target Ezh2 [25, 26]. Instead, miR-1, miR-133, and miR206 are specifically expressed in cardiac and skeletal muscle under the control of MyoD, SRF, and Mef2C transcription factors, and they regulate satellite cells proliferation and myoblast differentiation [23, 27, 28]. miR-208 and miR-499 are also specifically expressed in muscle and constitute a family of intronic miRNAs, referred to as “MyomiRs” [29]. These miRNAs are encoded by introns of the myosin genes and regulate myofibers specification. In addition, long noncoding RNAs (lncRNAs) are also emerging as regulators of the myogenic differentiation program [24, 30, 31]. Among lncRNAs, competing endogenous RNAs (ceRNAs) regulate miRNAs activity. Cesana et al., discovered that the muscle-specific lncRNA linc-MD1 acts as a natural decoy for miR-133 and miR-135, preventing them from binding to Mef2C and Mastermind-like 1 mRNAs and thus hampering miRNAs mediated negative effect on protein levels [24].

 In this paper we will focus on recent findings depicting the epigenetic landscape of myoblasts cells, obtained through the application of Next Generation Sequencing (NGS) technology. The most recent results presented by three independent research groups shed new light on the genomewide epigenome of myoblast cells, focusing on histone modifications signature, the genomewide location of the MyoD transcription factor and of chromatin modifiers, for example, PcG proteins.

## 3. The Next-Generation Sequencing “Revolution”: Brief Overview of NGS-Based Methods to Study Epigenomes

 In recent years, high-throughput technologies have been developed and rapidly improved to interrogate several aspects of cellular processes. Likewise, traditional approaches employed to interrogate the epigenome have undergone profound transformation since their coupling to massive parallel sequencing. As a result, approaches that investigated restricted groups of genomic loci have been adapted to genomewise analysis, with unprecedented resolution and specificity, and with dramatically decreasing sequencing costs.

Methodologies currently employed to investigate global epigenetic signature are summarized in Table 1 and briefly described as follows.

(i) 4C-Seq (circular chromosome conformation capture) is used to map long-range chromatin interactions and relies on proximity-based DNA ligation and sequencing of chromosomal regions contacting a bait DNA region [32].

Hi-C-Seq is also employed to define chromosome architecture but it is free of bias for a bait locus [33].

(ii) ChIA-PET-Seq (chromatin interaction through DNA-binding protein) combines chromatin immunoprecipitation (ChIP), proximity ligation, pair-end tag, and deep sequencing to detect chromatin interactions mediated by a protein, at the genomewide scale [34, 35].

(iii) MNase-Seq exploits the property of micrococcal nuclease (MNase) to preferentially cleave linker region DNA and the MNase-digested DNA can therefore be used to annotate nucleosomes distribution [36]. DNase sequencing on the other hand allows to map DNase I hypersensitive sites on the genome and therefore to identify accessible chromatin regions, which are not tightly compacted by nucleosomes [37]. FAIRE-Seq (formaldehyde-assisted isolation of regulatory regions) describes regulatory sequences with a reduced nucleosome content [38].

(iv) Chip sequencing (ChIP-Seq) couples the conventional chromatin immunoprecipitation technique with massive parallel sequencing, and it is used to profile histone modifications, and map transcription factors, core transcriptional machinery and chromatin modifiers recruitment in a genomewide scale. The genomewide map of generated reads provides information on the location and the intensity of the protein-DNA interaction [19, 39].

(v) Bisulfite-Seq relies on the ability of bisulfite to convert unmethylated cytosines to uracils so that massive parallel sequencing can identify cytosine methylation status at the single base pair resolution [40]. Alternatively, ChIP-based methods can be employed to enrich for methylated DNA, exploiting antibodies raised against 5-methylcytosine (MeDIP-Seq) [41] or methylated DNA-binding proteins (MBD-Seq) [42].

(vi) Furthermore, global mRNA expression profiling can now be achieved through a deep-sequencing approach (RNA-Seq) that sequences steady-state RNA (known and novel ones) in a sample, offering larger than microarrays dynamic range and sensitivity. However, a gene list is not the final step of this approach, deeper insight is gained by bionformatics resources such as gene enrichment analysis, pathways, and regulatory network analysis [43]. One key advantage of RNA-Seq over methodologies employed in the past is that it can profile mRNAs, lncRNA, and small RNAs at the same time.

## 4. Interpreting Transcriptional Regulation of Myogenesis

 Exploiting Chip assays coupled to massive parallel sequencing, Cao et al. identified the genomewide binding of MyoD, in C2C12 myoblast cells, satellite cells, and in mouse embryonic fibroblasts (MEFs) converted to myotubes by the stable expression of MyoD [44]. As predicted, MyoD is recruited to E-boxes of genes regulated during skeletal muscle differentiation. Unexpectedly, MyoD also constitutively binds additional nonmyogenic loci, with a high density of peaks in intergenic and intronic regions, in addition to an enriched distribution in promoter regions. Of note, the ChIP-Seq data presented show high concordance of results obtained from C2C12 myoblasts cells, primary satellite cells, and MEFs cells transduced with MyoD.

 Most of MyoD bound regions regulate genes expressed both in undifferentiated C2C12 and differentiated myotubes, while, in approximately 10% of MyoD targets, the binding is associated with distinct peaks in myotubes versus proliferating cells. Therefore, MyoD constitutively binds the majority of identified sites in both proliferating and differentiated C2C12 cells. Most of MyoD binding sites do not represent classical enhancer elements, since out of 25 MyoD bound regions only 10 showed enhancer activity in a transient transfection assay. Interestingly, MyoD broad binding throughout the genome correlates with chromatin regions with overall increase in their histone H4 acetylation levels in MyoD-transduced MEF cells versus control cells. Conversely, MyoD expression in MEF cells marginally impacts the distribution of the H3K4me3 marks. Thus, MyoD may play a crucial role in broadly reprogramming the epigenetic architecture of the lineage-specified cells. The latter findings suggest that MyoD association throughout the genome leads to the recruitment of histone acetyltransferases and most likely of chromatin remodeling factors [8], possibly independently of the local transcriptional regulation of the target region. Moreover, MyoD expression may deeply affect nuclear organization and genomic architecture. Early experiments in several cell lines demonstrated that MyoD is a master regulator able to convert certain cell types but not others to skeletal muscle [45, 46]. Therefore, MyoD ability to induce skeletal muscle-specific transcripts expression in nonmyogenic lineages may rely on its ability to associate a broad number of nonmyogenic target genes and to coordinate a broad reconfiguration of chromatin states. Further Hi-C-Seq experiments in MEF cells converted by MyoD overexpression and control MEF cells may reveal global spatial reorganization of the genome, triggered by MyoD-forced expression. Furthermore, ChIA-PET experiments may reveal MyoD-mediated long-range chromatin interactions [34, 35] and disclose how MyoD affects topological myoblast architecture.

## 5. Histone Modifications Signature in Myoblasts and Myotubes

 Asp et al. performed an initial comprehensive description of myoblasts and myotubes histone modifications profile [47]. The authors exploited the Chip technique coupled to massively parallel sequencing (ChIP-Seq) to identify the genomewide distribution of epigenetic marks and RNA polymerase II (RNA PolII). Data from ChIP-Seq experiments were merged with expression profiles data to depict a first portrait of epigenetic landscape changes underlying C2C12 myoblasts differentiation. The authors provide evidence that histone H3 acetylation at lysines 9 and histone H4 acetylation at lysine 12 are dramatically decreased on chromatin regions of differentiating myotubes, while H3K18Ac levels are reduced on constitutively active genes and on genes whose expression decreases in myotubes. Furthermore, promoters of genes upregulated in myotubes prematurely show enriched recruitment of RNA PolII and histone marks correlating with transcriptional activation in proliferating myoblasts, suggesting that they already acquire features of active chromatin, before they reach their maximal expression levels. In addition, the authors focus their attention on genes silenced both in myoblast and myotubes. Histone H3K27me3 is largely found in promoters, gene bodies, and intergenic regions. Genes marked by H3K27me3 show inverse correlation with RNA PolII recruitment. Genes marked by H3K27me3 modification can be divided into two classes: one group of genes displays H3K27me3 mark in undifferentiated state and is corresponding to a gene ontology (GO) category of genes essential for muscle formation. Consistent with previous studies [15, 22], a significant subset of these genes exhibits a reduction in H3K27me3 mark upon differentiation. Nevertheless, another subset of genes in this cluster is transcriptionally upregulated and is capable to maintain the H3K27me3 mark throughout differentiation.

 Furthermore, genes involved in nonmuscle lineage commitment can be grouped in a distinct cluster characterized by the H3K27me3 mark and a transcriptionally silent state, both in myoblasts and myotubes. Notably, mesoderm-related genes, such as genes involved in myogenesis, adipogenesis, and osteogenesis are not present in this cluster. Interestingly, as shown for other lineage committed cells [18], only a small fraction (11%) of H3K27me3 genes displays a bivalent mark in myoblasts.

 Because every cell lineage displays a specific genomewide profile of histone modifications, a comprehensive description of the chromatin signature of skeletal muscle cells will represent a precious resource for scientists investigating mechanisms underlying myogenic transcriptional regulation.

## 6. Chromatin Modifiers: Contrasting Roles of PcG Proteins in Myogenesis

 Genomewide mapping of chromatin factors has provided insights into the gene networks regulated by histone acetyltransferases, HDACs, and chromatin modifiers in ESCs and hematopoietic lineages [11, 48], which have been cellular systems at the forefront in the application of next-generation sequencing technologies (NGS). More recently, Mousavi et al. employed ChIP-Seq to investigate the genome-wide occupancy of chromatin modifiers belonging to the PcG proteins in skeletal muscle cells and explored their relation to RNA Polymerase II recruitment and the distribution of the opposing histone marks H3K27me3 and H3K4me3 [49]. As anticipated, Ezh2 recruitment substantially overlaps with the H3K27me3 marked genes in C2C12 myoblasts. Unexpectedly, Ezh1, an Ezh2 paralog that has been shown to have overlapping transcriptional repressive function in ESCs [50, 51], showed overlap with only 14% of H3K27me3 marked genes. Conversely, 51% of the regions occupied by Ezh1 are found to be associated with H3K4me3. Comparison of RNA-Seq profiles and RNA polymerase II global recruitment reveals that Ezh1 enriched regions are located on actively transcribed loci. Chip-Seq experiments with antibodies recognizing the elongating form of RNA Pol II (Phospho-serine-2 RNA Pol II) and a marker of elongation (H3K36me3) in Ezh1-depleted cells revealed that Ezh1 promotes RNA transcription, regulating the elongation step. Thus, genomewide mapping analysis unveiled a novel function for a member of PcG proteins, during the myogenic differentiation process.

## 7. Deep Sequencing Technologies and the Study of Skeletal Muscle Diseases

 NGS technologies open novel avenues in the study of myopathies: the new methodological approach will shed new light on the biological pathways involved in skeletal muscle diseases and become an essential tool to study genetic causes of myopathologies. Furthermore, it will likely become a valuable technological approach in molecular diagnosis [52].

 A recent report by Geng et al. highlights the role of ChIP-Seq technology in revealing the molecular pathways modulated by the homeobox protein DUX4, a candidate gene misexpressed in facioscapulohumeral dystrophy (FSHD) [53]. The authors performed chromatin immunoprecipitation assay followed by high-throughput sequencing in human skeletal myoblasts overexpressing the isoform of DUX4 (DUX4-fl) detectable in FSHD but not in control muscles. The set of genes identified as misregulated in DUX4-overexpressing cells has been also deregulated in FSHD skeletal muscle but not in control tissues, and this finding led authors to the identification of candidate pathways contributing to a better understanding of the disease etiology. Furthermore, this set of genes represents promising biomarkers to design novel diagnostic and treatment strategies.

 NGS approaches may also represent a suitable diagnostic tool for Duchenne and Becker muscular dystrophy, because of the heterogeneity in the mutational spectrum of the disease and the large size of the dystrophin gene [52]. Therefore, NGS-rooted technologies may not only be invaluable in the discovery of human disease genes but also represent an important avenue to be pursued in molecular diagnosis of myopathies in particular, as NGS protocols have been recently extended to analyze clinical samples that are formalin-fixed and paraffin-embedded [54].

## 8. Conclusions and Perspectives

 Comprehensive understanding of the myoblast/myotube epigenome will extend our fundamental knowledge of molecular mechanisms orchestrating biological processes, such as myogenic differentiation, muscle development, maintenance, and regeneration, in physiological and myopathological conditions. Deep sequencing methodologies will assist stem cell research toward the comprehension of mechanisms underlying muscle cells identity. This knowledge has important implication for regenerative medicine, underscoring new paradigms for satellite cells biology and offering novel targets of pharmacological intervention.

**Figure 1:**
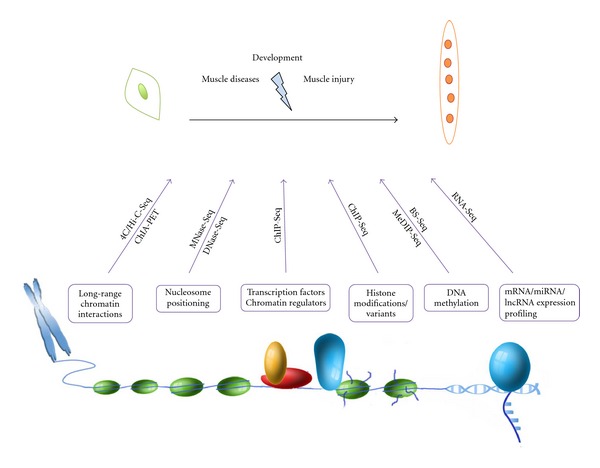
During development and muscle regeneration, satellite cells exit quiescence, actively proliferate, and terminally differentiate and fuse to form new muscle fibers. Within the nucleus, these steps are finely tuned by modifications in high-order chromatin structures and nucleosome accessibility, by changes in protein complexes recruited at regulatory regions, by alterations in histones marks and in the DNA methylation state. NGS offers invaluable means to explore each of these variations genomewide and to accurately identify pathways and regulatory networks underlying satellite cells activation and differentiation.

Recent advances in NGS technology allow addressing several epigenetic questions at the genomewide scale (Figure 1). To date, a restricted number of epigenetic regulators of myogenesis have been examined exploiting RNA-Seq and ChIP-Seq approaches. Although we predict that additional studies are underway, further studies are warranted to define global transcriptional targets and genomewide binding sites of transcription factors that modulate myogenesis (e.g., Pax7, Myf5, Myogenin, Mef2, SRF). A comprehensive description of their transcription targets and regulatory elements will define how individual transcription factors are connected to gene regulatory networks and how they influence each other at the genome level.

Characterization of the histone modifications landscape in skeletal muscle cells can be exploited further to annotate lineage-specific functional genomic elements, such as enhancers and insulators [55–57]. Chromatin profiling can therefore become a predictive means to define regulatory regions that are selectively functional, poised, or non-functional in myoblasts and myotubes or throughout mouse development. Moreover, the application of 4C/Hi-C-Seq technology and ChIA-PET in skeletal muscle cells that undergo differentiation will provide structural information on chromosomes long-range chromatin interactions and chromatin compartmentalization within the nucleus.

 Potential drawbacks of NGS techniques are the amount of starting material that frequently is a limiting factor. Isolation of quiescent satellite cells by cell sorting greatly reduces the number of cells one can use for RNA-Seq or ChIP-Seq experiments. Nevertheless, new protocols for chromatin immunoprecipitation assays coupled with massively parallel sequencing have been developed and optimized for limiting sample material [58]. Furthermore, third-generation sequencing platforms are now designed to sequence DNA at the single molecule and have already been used in proof of principle epigenetic studies [59].

 Another challenge introduced by NGS technology is the growing need for bioinformatics tools to analyze the vast amount of data generated by each experiment. Moreover, large-scale data obtained from NGS platforms need to be made available to the broad scientific community in a standardized and simple annotation form, as these data represent a precious framework to infer biological information. Bioinformatics pipelines require further refinements to enable expansion of mapping capabilities to the genome [60], improve normalization within and between samples to avoid high number of false positive differential results [61] and to better correlate results of ChIP-Seq, RNA-Seq, and whole-genome DNA methylation analysis at the single-nucleotide resolution.

 Besides implementing computational methods in biological studies, successful application of NGS technologies imposes a more profound revolution related to experimental design and forces a transition from single-gene hypothesis-driven experiments to a genomewide view, in which understanding of the integration and the cross-talk of different circuitries is at the forefront. The achievements of these remarkable research capabilities lie in the combination of conventional molecular and cellular biology approaches with a systemwide genetic view, which enables us to explore so far not addressable questions and will help us to gain a more comprehensive insight into mechanisms guiding skeletal muscle cells during differentiation, its maintenance and response to pathological insult.

## Figures and Tables

**Table 1:** 

Method	Method description	Genomewide data	Reference
4C-Seq, Hi-C-Seq	Chromatin proximity ligation and sequencing	3-Dimensional protein-DNA interaction Long-range chromatin interaction DNA looping	[23, 24]

ChIA-PET	Chromatin interaction analysis with paired-end tag sequencing: ChIP enrichment followed by chromatin proximity ligation and sequencing	Long-range chromatin interactions mediated by a DNA binding protein DNA looping	[25, 26]

DNase-Seq	Sequencing of DNaseI-digested DNA fragments	Chromatin accessibility and nucleosome positioning Identification of nucleosome-free regulatory regions	[28]

FAIRE-Seq	Formaldehyde-assisted isolation of regulatory elements sequencing	Chromatin accessibility Genome-wide mapping of protein-free DNA	[29]

MNase-Seq	Sequencing of micrococcal nuclease-digested DNA	Genomewide mapping of nucleosome-protected DNA nucleosome positioning	[27]

ChIP-Seq	Sequencing of DNA fragments obtained from chromatin immunoprecipitation assays	Genomewide mapping of protein-DNA interactions (a) Histone posttranslational modifications (b) Histone variants (c) Transcription factors (d) Chromatin modifiers and remodelers	[19, 30]

BS-seq	BS-Seq: bisulfite sequencing	DNA methylation	[31]

MeDIP-seq	MeDIP-Seq: methylated DNA immunoprecipitation sequencing	DNA methylation	[32]

MBD-seq	ChIP with methylated DNA-binding proteins	DNA methylation	[33]

RNA-Seq	Sequencing of RNA	Expression profiling Noncoding RNA expression Novel transcripts Alternative splicing	[34]
